# Using concurrent gait and cognitive assessments to identify impairments after concussion: a narrative review

**DOI:** 10.2217/cnc-2017-0014

**Published:** 2018-01-19

**Authors:** David R Howell, Michael W Kirkwood, Aaron Provance, Grant L Iverson, William P Meehan

**Affiliations:** 1 The Micheli Center for Sports Injury Prevention, Waltham, MA 02453, USA; 2 Division of Sports Medicine, Department of Orthopaedics, Boston Children's Hospital, Boston, MA 02115, USA; 3 Brain Injury Center, Boston Children's Hospital, Boston, MA 02115, USA; 4 Sports Medicine Center, Children's Hospital Colorado & University of Colorado School of Medicine, Aurora, CO 80045, USA; 5 Department of Physical Medicine & Rehabilitation, University of Colorado, Aurora, CO 80045, USA; 6 Rehabilitation Medicine, Children's Hospital Colorado, Aurora, CO 80045, USA; 7 Department of Physical Medicine & Rehabilitation, Harvard Medical School, Spaulding Rehabilitation Hospital, Boston, MA 02129, USA; 8 MassGeneral Hospital for Children Sport Concussion Program, Boston, MA 02114, USA; 9 Home Base, A Red Sox Foundation & Massachusetts General Hospital Program, Boston, MA 02129, USA; 10 Department of Pediatrics & Orthopaedic Surgery, Harvard Medical School, Boston, MA 02115, USA

**Keywords:** attention, dual-task, locomotion, mild traumatic brain injury, neurocognition, recovery

## Abstract

Understanding how a concussion affects an individual is oftentimes difficult for clinicians due to the varying symptom profiles reported by the patient and the multifaceted and heterogeneous nature of the injury. Accordingly, the interpretation of postconcussion performance can be challenging, because many different testing paradigms have been reported as potentially useful in the literature. Among the types of tests clinicians use to understand how concussion affects an individual, both gait and neurocognitive evaluations have demonstrated utility. Our purpose is to describe how combined gait and cognitive (i.e., dual task), as well as single-task gait and computerized neurocognitive examinations can assist clinical decision-making.

It is well established that concussions can have adverse effects on cognition [1], dynamic balance [2,3], static balance [1,4,5] and vestibular–oculomotor functioning [6–9]. People who sustain this injury report a diverse range of physical, cognitive and psychological symptoms [10–12]. Concussion is a multifaceted injury of the brain that affects individuals in a variable fashion [13]. Accordingly, it can be difficult to provide prognostic counsel to patients, or to determine the optimal time to resume school, work or athletic activities. Many different forms of testing have been proposed to contribute useful information to clinical evaluations after a concussion. These range from high expense and sophistication (e.g., advanced neuroimaging [14,15] and electroencephalography [16–18]), to moderate expense and sophistication (e.g., computerized neurocognitive tests [19–21]), to low expense and sophistication (e.g., Balance Error Scoring System [BESS] [22,23] and symptom inventories [24–26]). A proper balance of cost, time and added value often dictates the management practices within each setting, and leads to considerable variability among different practices throughout the USA [27,28].

Interpretation of postinjury performance can be challenging for many reasons. One potential reason is that questions remain pertaining to the natural history of concussion recovery [29,30]. Many of the effects of concussion, particularly concussions sustained during sports, resolve within 1–4 weeks postinjury for the majority of patients [4,31,32], resulting in a risk of false-positive findings after this time. Specifically, symptom inventories are nonspecific to concussion and may be affected by factors other than concussion-related dysfunction, such as exercise [33,34]. Other clinical tests that are commonly used in conjunction with symptom inventories possess limitations as well: the BESS is limited by practice, environmental and equipment effects [35–38], while computerized neurocognitive test performance may be influenced by sleep, prior history of concussion or attentional-deficit disorders [39–41]. Additionally, interpreting what constitutes ‘abnormal’ or ‘unusual’ performance can further complicate decision-making. Although comparison to baseline, or preinjury, performance remains the goal in many clinical situations [42], normative reference values on common concussion tests have been developed [43–50] and may allow clinicians to identify concussion-related deficits in the absence of baseline data [51]. As such, the purpose of this narrative review is to outline and discuss published studies that pertain to the clinical usefulness of dual-task gait in the management of sport-related concussion, as well as other tests of function, such as computerized neurocognitive tests or single-task gait examinations.

## Gait & posture

Both gait and balance evaluations are recommended as key features of a clinical concussion evaluation [52]. Although these terms are often used in conjunction, they both represent different aspects of postural control. Specifically, the term posture has been described as the orientation of the body relative to the vector of gravity, while balance describes how the body achieves stability during motion to avoid falling [53]. Therefore, gait occurs by maintaining an upright posture during bipedal locomotion. Gait is a relatively challenging task for the balance control system [53], and is a complex motor process that takes many years to develop during childhood [54]. No longer considered an automated motor output process, humans must be aware of, and appropriately control both limbs in space to appropriately produce gait [55].

Within the context of concussion management, balance assessments that rely on static control of posture are commonly used, while tests of dynamic motor abilities such as gait are not thought of as routine elements of a concussion examination [27,28,56]. Although no studies to this point have directly compared these two types of postural control deficits after a concussion, gait may involve a more complex set of motor actions required for successful completion and thus, a longer period of time required for recovery. Specifically, measurements like gait speed, stride length and interjoint coordination have contributed useful information when assessing deficits resulting from a concussion [57–59], while more in-depth measures of center of mass (COM) movement may allow researchers to understand fine motor control processes of the body and potentially identify subtle pathologic gait abnormalities after concussion [3,60,61].

## Neurocognitive function

Numerous studies have documented that concussion can cause impairment across a variety of cognitive domains including new learning and memory, processing speed, attention and executive functioning [1,11,21,62–64]. Deficits are observed whether a comparison is made to an athlete's pre-injury (baseline) status or a control group's performance [51]. The evaluation of cognition can be achieved through sideline day-of-injury mental status screening [65] or more thorough neuropsychological testing [66]. Accordingly, both have advantages and demonstrated value in helping to objectively identify acute injury effects.

Objective sideline cognitive screening (e.g., using the standardized assessment of concussion [5,31]), which can be administered by any trained professional and takes only a few minutes to complete, is more sensitive to concussion than informal orientation questions [5]. Lengthier neuropsychological testing is administered via traditional paper-and-pencil measures or by computer. In the sports setting, such testing is often employed in a manner consistent with the ‘baseline testing’ model originally proposed in the 1980s [67,68]. The model involves athletes undergoing a test battery during a preseason baseline session and then repeated follow-up testing after a concussion until the athlete returns to the baseline performance. At this point, computerized neurocognitive tests are the most common form of objective testing used in sports medicine settings: approximately 30% of universities [27] and 40% of high schools [21] use some form of a computerized neurocognitive test.

## Dual tasks

Although cognitive abilities are commonly assessed as a part of concussion management plans, concurrently pairing a cognitive and motor task such as gait together may allow for detection of deficits that are not identified through single-task gait or cognitive testing alone. Gait has been traditionally thought of as an automated motor process, but it is now understood that multiple neurophysiological influences may modulate walking behavior [55]. In healthy individuals, gait can be modulated by perceived physical or cognitive perturbations that force the division of attention while walking. This process of combining two forms of testing has been termed as dual task and has been used traditionally to assess health-status progression among aging populations at risk for falling [69–71]. Although researchers have observed considerable concussion-related dual-task impairments [2,72], they are not currently used in a widespread fashion during clinical examinations [57]. Although single tasks, such as evaluations of gait, quiet stance or neurocognitive function in isolation, require the focus of attention, dividing attention across multiple domains leads to the competition for resources. In the injured brain, this competition may result in the degradation in one or both of the domains when completed concurrently relative to an uninjured and otherwise healthy individual completing the same type of task [3].

Attentional deficits persist in some people after a concussion. Specifically, while the spatial orientation component of attention may be altered initially after injury [64], deficits in the executive component of attention (so called ‘executive functions’) may persist among both adolescents and young adults for a longer time period than has been typically measured using traditional clinical metrics [5,63,64]. Therefore, due to the reduction in attentional resources after a concussion, dual tasks may be well-suited to identify persistent impairments.

Although many dual-task concussion studies have focused on a gait and cognitive task performed concurrently, other methods have been used to examine the simultaneous completion of different functional abilities. For example, Tapper *et al*. implemented a dual-task paradigm that combined an auditory discrimination task and a Corsi block task simultaneously [72]. They observed that those who reported a history of concussion had a more difficult time dividing attention between the two tasks relative to their peers with no concussion history. In addition, Resch *et al*. measured cognitive abilities while balance control was quantified in various static positions among healthy collegiate students, observing that postural control performance was prioritized over cognitive processing [73]. Furthermore, during the BESS test, the addition of a secondary cognitive task appears to improve reliability and clinical applicability within concussion evaluations [74].

Among the many current challenges for researchers and clinicians interested in potential treatments for concussion is the lack of an objective diagnostic technique and method to more accurately quantify physiologic recovery of the brain. If such measures were developed in a manner that was adaptable for widespread use, assessment of the efficacy of different concussion treatment pathways might be easier to determine. Dual-task function after a concussion, therefore, constitutes the combination of two aspects that may assist in outlining and evaluating the efficacy of future randomized control treatment or rehabilitation trials.

As our primary area of review pertained to the use of dual-task methodology (i.e., combined cognitive and gait tests) to evaluate individuals after a concussion, we conducted a review according to the Preferred Reporting Items for Systematic Reviews and Meta-Analyses (PRISMA) guidelines [75], using the following search engines: PubMed, Web of Science and Academic Search Premier (EBSCO host) from the time of database inception to January 2017. Specific search terms included sports, concussion, mild traumatic brain injury, gait, locomotion, dual task*, concurrent task* and simultaneous task*. Asterisks were used at the end of a search term to identify all terms beginning with that string. Reference lists from identified articles were also used as a supplemental search technique. All titles and associated abstracts were reviewed independently and publications that were not appropriate were excluded from the review.

## Single-task gait evaluations

The measurement of single-task gait, or normal walking behavior, has been studied extensively from a neuromuscular and kinematic perspective [76–78]. In the context of body mechanics, humans walk in a relatively unstable fashion whereby the COM of the whole body regularly extends outside of the base of support area [76]. As such, bipedal locomotion requires complex neuromuscular control to prevent falling and to achieve and maintain stability. Despite the complexity of this process, gait patterns are highly consistent across time in uninjured young adults and adolescents [79]. After a concussion, this fine neuromuscular control ability may be impaired. As a result, those with a recent concussion often walk slower or with shorter strides than uninjured control counterparts during single-task gait [80–82]. Therefore, the interpretation of single-task gait behavior may provide some value in the context of concussion management.

After a concussion, sophisticated measurements of steady-state walking behavior have been useful in identifying movement pattern deficits. Using a pressure mat to measure spatio-temporal gait parameters, more conservative gait characteristics were observed in those with a history of concussion, indicated by shorter stride lengths and slower step velocities [83]. The authors suggested that this may be a result of persistent postconcussion postural control deficits that go undetected with other traditional forms of concussion testing or that it may be an early indicator of potential long-term neurological impairments resulting from multiple concussions. Future prospective cohort studies, however, are needed to adequately determine the cause of this observation, but these findings suggest that gait alterations may be more apparent in those with a history of multiple concussions.

The length of time that individuals with a concussion exhibit single-task gait deficits is not currently well defined. Prior research suggests that group differences between those with a concussion and matched controls exist from the time of injury and until approximately 2 weeks after injury [3,84]. This timeline coincides with the duration of cognitive test or symptom recovery time after concussion previously reported [31,32]. The benefits of examining dynamic balance control in addition to standard spatio-temporal variables during gait appear to be relevant in the identification of postconcussion deficits as well. Conservative gait patterns indicative of dynamic instability can be quantified by COM movement, or the interaction between the COM and the base of support during gait [85]. The location of where each foot is placed during gait may reflect the control of the whole body COM and the ability to dynamically control the body during movement [85]. Thus, neuromuscular deficits after a concussion may arise from motor control difficulties responsible for controlling where each foot is placed during locomotor progression.

The apparent inability to properly control the mass of the body during gait progression can result in more side-to-side movement (or sway) of the COM. Therefore, this variable has been used consistently to identify dynamic stability [60,85–87]. Using motion analysis, differences in gait between injured and control groups remain on measures of dynamic balance, even after there are no longer differences in self-reported symptom scores [3,84]. Although single-task gait abnormalities may exist after a concussion, quantification is difficult in most settings. Because balance testing is recommended as a part of the postconcussion evaluation, few collegiate athletic settings routinely use something other than the Balance Error Scoring System (BESS) or modified BESS (mBESS) [27,56].

## Neurocognitive evaluation

The evaluation of neuropsychological deficits after concussion has been reported throughout the literature, and has been reviewed in depth previously [88–90]. Their use in multifaceted concussion evaluations can provide helpful information regarding recovery from injury, particularly when test results are interpreted by a qualified neuropsychologist [52]. As mentioned previously, two methods are commonly used in the setting of sports concussion to evaluate cognitive functions: brief sideline screening and lengthier neuropsychological testing. Acute screening of mental status within hours of a suspected concussion may be one way to assist with concussion diagnosis [1,31,91] and is widely agreed to be important for assisting with immediate management [42,92,93]. Lengthier neuropsychological testing in the baseline model was once viewed as the ‘cornerstone of concussion evaluation’, [66,94] primarily because of its intuitive appeal, objective nature and resultant agreement statements from a number of professional organizations [42,92,93,95].

Practical advantages of baseline computerized testing are frequently promoted to justify its use including the ability to test many athletes simultaneously during the preseason, widespread availability, ease of administration and scoring, access to alternate test forms and ready creation of centralized data repositories. Computerized cognitive testing in the acute period may also be helpful for identifying athletes at risk for slower than expected recoveries [96,97] and identifying difficulties in athletes who no longer report symptoms [98]. Specifically, Broglio *et al*. identified that once asymptomatic after concussion, approximately 38% of athletes continued to demonstrate a deficit in at least one domain of neurocognitive function, such as verbal memory, visual memory, visual-motor speed or reaction time [98]. At the same time, concerns have been expressed that the promotion of computerized tests has outpaced their clinical evidence [99,100]. Despite these concerns, computerized neurocognitive tests possess clinical value in ability to recognize potential deficits beyond symptom inventories, particularly when used in the first week after a concussion [101].

Increased scrutiny of the empirical literature over the last decade has led to questions about the clinical utility of baseline testing as used popularly. Testing an athlete during the preseason requires time, effort and money. Although the collection of baseline data is ideal, recent studies suggest that neurocognitive data may be useful, even in the absence of baseline data, by comparing to normative values [51,102,103]. Other questions relate to whether the available tests have robust enough psychometric properties to allow for the accurate detection of change within the context of modest-to-poor test–retest reliability. Some studies have found poor test–retest reliabilities [99,100]. A recent study compared three commonly used computerized cognitive batteries (ANAM, Axon Sports/Cogstate Sport and ImPACT) and found that only about a quarter of the test indices had moderate stability coefficients (r >0.70) [101]. Moreover, although the tests were found to be reasonably sensitive to concussion within the first 24 h of injury, the sensitivity was near the false-positive rate established by nonconcussed athletes by 8 days postinjury, due in large part to clinical recovery.

Baseline cognitive testing has some methodological challenges and limitations. First, there is some evidence that athletes tested in a group setting perform more poorly on computerized cognitive testing than those tested individually [104], and they also have a somewhat greater rate of invalid scores [104] – but not all studies have found a difference associated with small group testing [105]. Deliberate underperformance on baseline testing in an effort to ‘sandbag’ has received limited attention given the fact that sandbagging has been found to occur in anywhere between 13 and 26% of athletes [106], although properly trained test administrators are usually able to detect such sandbagging [107]. How frequently an athlete should undergo baseline testing is another question without clear empirical guidance, particularly for children and teens who are still undergoing active cognitive development. A number of individual factors have also been found to affect neuropsychological test performance, which could influence whether an athlete is considered recovered from a concussion or not. For example, youth with attention-deficit/hyperactivity disorder [108,109] or learning disorders [108] perform more poorly on some baseline tests. Moreover, a large number of variables can influence symptom reporting on questionnaires that are often given during baseline testing, such as attention-deficit/hyperactivity disorder, learning disabilities, mental health difficulties, somatization and insufficient sleep [39,110–112].

These complexities render interpretation of neurocognitive tests more challenging than some might believe based on the seemingly straightforward ‘red light, green light’ approach produced by a number of computerized neurocognitive batteries. As such, neuropsychologists are now recognized as the best trained professionals to interpret neurocognitive test results, given expertise in psychometrics and knowledge of both brain injury and nonbrain injury effects on cognitive test performance [92]. In the end, neurocognitive testing can contribute useful clinical information after concussion. It can be particularly helpful when integrated into a more comprehensive multidimensional postconcussion assessment. Of note, because the etiology of persistent symptoms after concussion often involves neurologic and psychosocial factors, neuro-psychological evaluation by a neuropsychologist is recognized as important in disentangling injury and noninjury effects [92,113] and may even be useful as an intervention itself for reducing persistent symptoms [114].

## Dual-task gait

As discussed previously, many concussion evaluations probe single attention-focused abilities, such as quiet stance tasks, gait tasks or computerized neurocognitive tasks. Although these forms of assessment are a valuable component of the multifaceted concussion testing battery [42,52,92], combining both cognitive and motor function paradigms into a single assessment paradigm provides a method to increase task complexity, and potentially to identify deficits after concussion that are not detected with single-task assessments [84]. The ability to divide attention across multiple domains may be indicative of real-life activities. Because attention can be impaired after concussion [63,64], completing two competing tasks simultaneously may be more difficult for those who recently sustained a concussion compared with those who did not. Therefore, dual-task paradigms may be uniquely equipped to assess functional recovery after a concussion. Specifically, gait deficits during dual-task conditions appear to be more apparent compared with single-task conditions [3,82,84,115].

The test characteristics of dual-task gait paradigms have not been well-established to this point, particularly compared with more researched tests of neurocognitive function. Instead, much of the literature has focused on the comparison of acute postconcussion performance with matched controls within adolescent and young adult athletic populations. Questions remain about whether dual-task gait paradigms have adequate reliability, validity and sensitivity to be useful in clinical practice. Two recent studies have reported medium-to-high test–retest reliability for dual-task gait paradigms among healthy young adults [116] and healthy young adults and adolescents [79]. Specifically, when tested approximately 6 months apart, uninjured collegiate athletes had acceptable dual-task gait intraclass coefficient values ranging from 0.73 to 0.85 [116]. When adolescents were tested on five separate occasions over the period of 2 months, they also had acceptable levels of test consistency on dual-task gait balance control measures (Cronbach's α range = 0.79–0.96). Therefore, dual-task gait appears to be at least a reliable method for repeat administration among uninjured athletes (Table 1).

**Table 1.** **Dual-task gait characteristic variables that have been reported to assist with detecting postconcussion deficits.**

**Gait characteristic**	**Postconcussion deficits**	**Reliability**
Average walking speed	Slower among those with concussion than controls [122] Higher dual-task costs compared with controls [3,82]	Cronbach's α = 0.94–0.96 [79] ICC = 0.77 [116]

Stride length	Shorter after recent concussion in those with history of multiple concussions than controls [123]	Cronbach's α = 0.90–0.91 [79] ICC = 0.73 [116]

Double-leg stance support time	Greater among those with a history of concussion compared with those without a history of concussion [124]	Not available

COM medial-lateral displacement	More side-to-side sway within 48 h of injury during dual-task gait compared with controls [81] More side-to-side sway throughout the 2 months after concussion compared with single-task gait [3,82]	Cronbach's α = 0.79 [79]

COM peak medial-lateral velocity	Faster side-to-side movement during dual-task gait than single-task gait after concussion [3,82]	Cronbach's α = 0.85–0.89 [79]

Note: Reliability data was calculated using Cronbach's α across several longitudinal time points or ICCs at two distinct longitudinal time points.

COM: Center of mass; ICC: Intraclass coefficient.

Several investigations have observed that when gait and cognitive tasks are performed simultaneously, athletes with a concussion display deficits not otherwise detected. Within 48 h of concussion, collegiate athletes display greater COM sway than matched controls during a dual task consisting of spelling five-letter words in reverse, subtraction by sevens or reciting the months of the year in reverse order while walking [81]. No between-group differences, however, were identified during the walking only condition. Similarly, while other measures of dynamic balance control such as the peak velocity of the COM (a measurement taking into account both stride length and stride time) revealed initial postinjury deficits, no single-task differences were found between concussed and control groups across a 1 month postinjury timeline [117].

From the time of injury and throughout the ensuing recovery, athletes have also consistently shown greater deficits during dual-task walking relative to single-task walking after a concussion [3,118]. Some adolescent athletes walk with greater dual-task medial-lateral COM sway throughout a 2-month period after concussion relative to their own single-task performance and to the dual-task gait characteristics of matched controls [3]. Furthermore, the specific type of cognitive task that an individual performs during gait influences the amount of dynamic instability after a concussion, where more complex cognitive tasks are associated with greater dynamic instability [84]. Although Fino observed higher dual-task costs (i.e., the relative change from single-task to dual-task gait measures) among athletes after a concussion for gait speed, stride time variability or local dynamic stability [82], single-task gait speed differences were also detected between concussion and control groups. The degree to which these differences were observed varied by task, however, the addition of an arithmetic task during gait generated a greater destabilizing effect on those with a concussion. This was interpreted to suggest that while single-task gait may provide some beneficial information about recovery from concussion, dual tasks disrupt gait patterns and stability of those with concussion to a greater degree than single-task assessments [82].

Collectively, these studies demonstrate that across different age groups, testing timelines after concussion, and measurement devices, dual-task deficits are more apparent and continue to persist longer postconcussion than single-task gait deficits. This has implications for clinical practice, because many commonly used testing methods rely primarily on single-task paradigms. Using single-task paradigms alone may result in clinicians not being able to identify subtle, yet potentially meaningful neuromuscular control deficits among athletes prior to resumption of full athletic participation. Because dual-task testing may be more representative of the demands of sport than single-task tests, their inclusion during postconcussion examinations may help to identify those potentially at risk for another injury that may be missed using standard measures.

### Return to play

One area of recent interest to concussion researchers has been identifying impairments that continue to persist beyond clinical recovery when defined using ‘traditional’ measures (e.g., symptom burden returned to zero or baseline, normal or baseline neurocognitive test scores and normal or baseline BESS scores). This area of investigation is highly relevant to clinicians involved in the management of sport-related concussion, as accurate identification of when an individual has fully recovered from injury is an essential aspect of determining when it is appropriate for them to return to athletics. Although many studies have continued to follow athletes after concussion until they resume full athletic participation, few have continued to examine the effects beyond this point in time. Electroencephalographic evidence indicates that some individuals continue to exhibit neurophysiological differences a week after concussion, despite performing at a similar level as controls on traditional neurocognitive tests [16]. Such a phenomenon may underlie the ability for an athlete to ‘pass’ the traditional battery of clinical concussion tests yet still be experiencing physiologic dysfunction.

Using motion analysis, Parker *et al*. observed a 20% increase of COM movement (i.e., worsening neuromuscular control) among participants between 2 weeks and 1 month after concussion, while all had been cleared to return to play within the first 2 weeks of injury [119]. The authors hypothesized that the combination of incomplete recovery and return to full sports too soon after injury may have led to these changes, and that medial-lateral COM sway during dual-task gait may be a particularly sensitive variable to neuromuscular function. Similarly, elite athletes tested approximately 37 days following a concussion who did not present with postconcussion symptoms and scored similarly as controls on a battery of neuropsychological tests still showed navigational deficits in complex environments during gait [120]. Furthermore, among a sample of children approximately 43 days after a concussion who reported recovery of symptoms, single-task gait stride widths were significantly higher than controls [121]. The authors theorized that trouble integrating sensory information may have resulted in difficulty generating an appropriate motor output during tasks that require adequate neuromuscular function. No dual-task gait deficits were detected in this study, however, which the authors attributed to the use of a cognitive test during gait that was not complex enough to identify between-group differences [121]. Finally, using a prospective design, an altered course of recovery was found on dual-task gait balance control measures before and after athletes were cleared to fully resume pre-injury levels of athletic participation [118]. Although improvements were found immediately prior to resumption of athletic activities among all measurements, a worsening occurred in the ability to maintain dynamic stability during dual-task gait after resuming full participation in athletic activities. In conjunction, no changes were found after athletic activity resumption for single-task gait, symptom severity or computerized attention measurements. Thus, these findings further support the notion that although athletes are able to pass the clinical battery of tests and subsequently return to play, they may still be experiencing functional deficits.

Although dual-task gait paradigms appear to possess utility for concussion management protocols, the literature to date remains limited in many aspects. As mentioned previously, dual-task gait paradigm test characteristics have yet to be firmly established. Furthermore, few studies have investigated athletic populations younger than high school or older than college aged. One study of children who were an average age of 13 years observed dynamic single-task deficits compared with controls, but was conducted in a cross-sectional manner, so the length of time required for this younger population to recover after concussion remains difficult to determine [121]. Among older individuals, a group of elite athletes were tested at a similar postinjury testing time and also, despite no presence of clinical deficits, individuals with a concussion displayed dual-task gait under complex navigational environments [120]. As few studies have investigated how age affects gait balance control recovery after concussion [61], more work is needed to directly understand age group recovery differences during single-task and dual-task gait.

## Conclusion

Gait characteristics and cognitive abilities are affected by concussion. Although assessing each of these domains in isolation is useful in evaluating an athlete with a concussion, the simultaneous execution of both a gait and cognitive task may induce additional complexity that allows for the identification of impairments beyond those identified with traditional concussion tests. Thus, each of these different test forms can contribute meaningful clinical information after a concussion, and should be used within multifaceted postconcussion assessments based on the needs and limitations for each clinical setting.

**Executive summary**

- Many objective tests have been developed to assist with clinical decision-making following concussion.
- No single test will provide perfect sensitivity and specificity to identify all concussion-related deficits.
- Dual-task gait tests combine a cognitive and balance test simultaneously, potentially allowing clinicians to detect subtle concussion-related impairments.
